# Current and Projected Heat-Related Morbidity and Mortality in Rhode Island

**DOI:** 10.1289/ehp.1408826

**Published:** 2015-08-07

**Authors:** Samantha L. Kingsley, Melissa N. Eliot, Julia Gold, Robert R. Vanderslice, Gregory A. Wellenius

**Affiliations:** 1 Department of Epidemiology, Brown University School of Public Health, Providence, Rhode Island, USA; 2 Rhode Island Department of Health, Providence, Rhode Island, USA

## Abstract

**Background:**

Climate change is expected to cause increases in heat-related mortality, especially among the elderly and very young. However, additional studies are needed to clarify the effects of heat on morbidity across all age groups and across a wider range of temperatures.

**Objectives:**

We aimed to estimate the impact of current and projected future temperatures on morbidity and mortality in Rhode Island.

**Methods:**

We used Poisson regression models to estimate the association between daily maximum temperature and rates of all-cause and heat-related emergency department (ED) admissions and all-cause mortality. We then used downscaled Coupled Model Intercomparison Project Phase 5 (CMIP5; a standardized set of climate change model simulations) projections to estimate the excess morbidity and mortality that would be observed if this population were exposed to the temperatures projected for 2046–2053 and 2092–2099 under two representative concentration pathways (RCP): RCP 8.5 and 4.5.

**Results:**

Between 2005 and 2012, an increase in maximum daily temperature from 75 to 85°F was associated with 1.3% and 23.9% higher rates of all-cause and heat-related ED visits, respectively. The corresponding effect estimate for all-cause mortality from 1999 through 2011 was 4.0%. The association with all-cause ED admissions was strongest for those < 18 or ≥ 65 years of age, whereas the association with heat-related ED admissions was most pronounced among 18- to 64-year-olds. If this Rhode Island population were exposed to temperatures projected under RCP 8.5 for 2092–2099, we estimate that there would be 1.2% (range, 0.6–1.6%) and 24.4% (range, 6.9–41.8%) more all-cause and heat-related ED admissions, respectively, and 1.6% (range, 0.8–2.1%) more deaths annually between April and October.

**Conclusions:**

With all other factors held constant, our findings suggest that the current population of Rhode Island would experience substantially higher morbidity and mortality if maximum daily temperatures increase further as projected.

**Citation:**

Kingsley SL, Eliot MN, Gold J, Vanderslice RR, Wellenius GA. 2016. Current and projected heat-related morbidity and mortality in Rhode Island. Environ Health Perspect 124:460–467; http://dx.doi.org/10.1289/ehp.1408826

## Introduction

A large body of evidence has linked extreme heat events (i.e., “heat waves”) with higher mortality, especially from respiratory and cardiovascular diseases (Basagaña et al. 2011; D’Ippoliti et al. 2010; Kaiser et al. 2007; Le Tertre et al. 2006). Similarly, across a broader range of temperatures, higher “warm” temperatures have been consistently associated with higher mortality (Basu and Samet 2002; Basu et al. 2008; Curriero et al. 2002; O’Neill et al. 2003; Petkova et al. 2014; Stafoggia et al. 2006). Interestingly, the reported associations between moderate or extreme heat and morbidity have been less consistent (O’Neill and Ebi 2009), with most studies failing to find an association with cardiovascular morbidity (Bhaskaran et al. 2010; Green et al. 2010; Gronlund et al. 2014; Kovats et al. 2004; Mastrangelo et al. 2007; Michelozzi et al. 2009; Williams et al. 2012a, 2012b), but several studies reporting an association with health care utilization for renal diseases (Basu et al. 2012; Fletcher et al. 2012; Green et al. 2010; Gronlund et al. 2014; Hansen et al. 2008; Knowlton et al. 2009; Kovats et al. 2004; Li et al. 2011; Williams et al. 2012a). Many of these studies have focused on extreme heat, leaving greater uncertainty about the potential effects of moderate heat—which by definition occurs more often than extreme heat—on morbidity, and which individuals may be at greatest risk of these effects. Additionally, few studies have estimated the effects of higher temperatures on both mortality and morbidity together, leaving open the possibility that differences in associations between heat and morbidity and mortality are attributable at least partly to differences in study methods, data quality, or locations studied. Finally, few studies have specifically considered the association between present-day temperatures and morbidity in New England, despite suggestions that the adverse health effects of excess heat may be particularly pronounced in this area of the United States (Anderson and Bell 2011; Wu et al. 2013).

The Intergovernmental Panel on Climate Change (IPCC) has concluded that global warming is “unequivocal,” as evidenced by higher global average land and ocean surface temperatures, higher rates of ice melting, rising global mean sea levels, and higher atmospheric concentrations of greenhouse gases, and that it is “extremely likely” predominantly attributable to human influence (IPCC 2013). Climate models project further increases in surface temperature globally, and previous studies have projected that higher latitudes and northeastern coastal U.S. states will have greater future heat-related adverse health impacts than other regions (Luber and McGeehin 2008; McGeehin and Mirabelli 2001; Wu et al. 2013).

National, state, and local public health and emergency management agencies need a clear understanding of the health risks posed by excess heat, now and under the warmer temperatures projected for the future (O’Neill and Ebi 2009). Accordingly, the goals of this study were to *a*) quantify the association between maximum temperature and emergency department (ED) visits and mortality in Rhode Island, *b*) identify characteristics that may place individuals at greater risk of adverse health effects, and *c*) estimate the excess morbidity and mortality that would occur if this population were exposed to the warmer temperatures projected through the end of the century.

We evaluated these goals using population-based data from the state of Rhode Island, a small New England state with a land surface area of 1,045 mi^2^, 384 mi of shoreline, and a climate consisting of four seasons with an average annual temperature of 50°F. Rhode Island typically experiences a more moderate climate than other New England states because of its proximity to the Atlantic Ocean, but can also experience sudden changes in weather because it is located at the intersection of several storm tracks (State of Rhode Island 2014).

## Methods

*Exposure assessment.* We obtained data on temperature and dew point from 52 stations in Massachusetts, Connecticut, and Rhode Island for the study period from the National Oceanic and Atmospheric Administration’s (NOAA) Integrated Surface Database (NOAA 2014). We obtained data on fine particulate matter [PM ≤ 2.5 μm (PM_2.5_); *n* = 40 stations] and ozone (O_3_; *n* = 44 stations) air pollution from the Environmental Protection Agency from stations in Rhode Island, Massachusetts, and Connecticut [U.S. Environmental Protection Agency (EPA) 2014].

Because most of the weather stations in Rhode Island are near the coast, we used ordinary kriging methods [geoR package in R version 3.0.0 (R Core Team 2013)] to interpolate the daily maximum temperatures for the geographic centroid of the state (41.68364°; –71.52878°). For consistency in methods, we similarly used ordinary kriging to interpolate PM_2.5_, O_3_, and dew point for the geographic centroid of the state. These kriging models had a 10-fold cross-validated *R* ^2^ of 0.92 for maximum daily temperature, 0.85 for dew point, 0.80 for PM_2.5_, and 0.73 for O_3_, and no days with missing predictions.

*Outcome assessment.* We obtained from the Rhode Island Department of Health individual-level data on all ED visits between 2005 and 2012 to Rhode Island hospitals, excluding the Veterans Affairs Hospital and psychiatric hospitals, and data on all deaths in the state from 1999 through 2011. Data available on each ED visit included sex, age, race, health insurance information, admission date, census tract of residence, and primary and secondary discharge diagnoses using *International Classification of Diseases, 9th Revision* (ICD-9) codes. Mortality records included information on sex, age, race, census tract of residence, date of death, and primary and secondary cause of death using ICD-10 (*10th Revision*) codes.

We defined all-cause ED admissions as patients admitted to the ED with a primary discharge diagnosis code of ICD-9-CM < 800, E900, or 992. Similar to prior studies (Gronlund et al. 2014), we additionally identified ED admissions specifically related to heat (ICD-9: E900, 992), dehydration (ICD-9: 276.51), cardiovascular disease (ICD-9 390–429), renal disease (ICD-9 580–589), acute renal failure (ICD-9: 584), respiratory disease (ICD-9 480–487, 490–492, 494–496), and asthma (ICD-9 493). Admissions were considered to be heat-related if heat or dehydration were identified as either a primary or secondary discharge diagnosis. We defined all-cause mortality as all deaths including heat-related injury (ICD-10 T67) but excluding all other external injury-related deaths (ICD-10 S, T, V, and Y codes). The associations between heat and specific causes of death were not considered as there were insufficient deaths.

*Projection of future temperatures.* We obtained projections of daily maximum temperature at the geographic centroid of Rhode Island for 2046–2053 and 2092–2099 using the downscaled Coupled Model Intercomparison Project Phase 5 (CMIP5) multi-model ensemble projections (Maurer et al. 2007). CMIP5 is the most recent aggregation of climate change model experiments designed to project near-term and longer-term climate worldwide. CMIP5 projections use four representative concentration pathways (RCPs), each of which assumes different trajectories for greenhouse gas emissions and other forcings (Brekke et al. 2013). We chose RCP 8.5 and RCP 4.5 because they represent scenarios with relatively higher and lower emissions, respectively, therefore providing a range of possible future scenarios. Specifically, the RCP 8.5 scenario is characterized by increasing greenhouse gas emissions over time (van Vuuren et al. 2011), whereas RCP 4.5 assumes that technological improvements and policy incentives will reduce future greenhouse gas emissions (Thomson et al. 2011). The daily bias-correction and constructed analogs (BCCA) downscaled CMIP5 multi-model ensemble includes daily maximum temperature projections from 42 coupled ocean–atmosphere circulation models for the RCP 4.5 emissions scenario and output from 41 models for the RCP 8.5 emissions scenario (see Supplemental Material, Table S1). The spatial resolution of the downscaled daily temperature projections was 1/8°.

*Statistical analyses.* As in prior studies (e.g., Anderson et al. 2013), we used overdispersed Poisson regression models to estimate the association between daily maximum temperature and rates of all-cause and heat-related ED admissions and all-cause deaths. We modeled same-day daily maximum temperature using a natural cubic spline with 3 degrees of freedom to allow for a nonlinear exposure–response relationship. In secondary analyses we additionally considered cause-specific ED admissions with a primary discharge diagnosis of respiratory disease, cardiovascular disease, renal disease, acute renal failure, asthma, and heat (not including dehydration). All models were adjusted for day of week (a factor with seven levels), federal holidays (a dichotomous variable that is true for federal holidays and false otherwise), 8-hr maximum O_3_ (a continuous variable representing the daily maximum 8-hr O_3_ concentration in ppm), dew point (modeled as a natural cubic spline with 3 degrees of freedom), and seasonal and long-term time trends (modeled as natural cubic splines with 5 degrees of freedom per year). In sensitivity analyses we additionally adjusted for PM_2.5_ modeled as a continuous variable representing the daily mean concentration (micrograms per cubic meter). Additional analyses stratified by age group (< 18, 18–64, ≥ 65 years), race (white vs. non-white), and sex were performed with respect to all-cause ED admissions and heat-related ED admissions.

For each model, we report the overall *p*-value comparing by analysis of variance (ANOVA) the full model to the same model without any terms for temperature. This *p*-value corresponds to the omnibus test of whether temperature (as modeled) is a statistically significant incremental predictor of the outcome. For each model we used standard methods to calculate the percent change in the outcome [and 95% confidence intervals (CIs) and *p*-values] associated with specific 10-degree increments in maximum daily temperature (Rothman 2012). If the exposure–response relationship was perfectly linear, then the incidence rate ratio would be the same for any 10-degree increment within the range of the data. But for nonlinear exposure–response relationships, the association per 10-degree increment in maximum temperature differs depending on where along the exposure–response curve one starts. The *p*-value for a particular 10-degree shift in maximum temperature tests the null hypothesis that this specific shift in maximum daily temperature is not associated with the outcome, adjusting for the potential confounders included in the model. This increment-specific *p*-value may differ from the omnibus *p*-value because the latter is a test of the overall association between daily maximum temperature across the entire temperature range and the specific outcome. Exposure–response curves are described as approximately linear or not based on subjective assessment, but results are presented both graphically and numerically to allow readers to judge for themselves.

We projected the number of additional all-cause ED admissions, heat-related ED admissions, and deaths that would be observed if the Rhode Island population of 2005–2012 was exposed to the higher maximum temperatures projected for April–October of 2046–2053 and 2092–2099. Specifically, we used the daily maximum temperatures projected for each of 42 climate models for the RCP 4.5 emissions scenario and the 41 climate models for RCP 8.5 emissions scenarios for the years 2046–2053 and 2092–2099 to predict future morbidity and mortality based on the fitted model coefficients from the analyses of data from 2005 to 2012 (or 1999 to 2011 in the case of deaths), with the equation

where *S*(^·^) represents a smooth function of (^·^) modeled using natural cubic splines, *Temp* denotes projected future daily maximum temperature, *Time*, *Dewpt*, *Ozone*, *Holiday*, and *DOW* denote the values of time, dew point temperature, O_3_, holidays, and day of week, respectively, observed during the reference period and ^ˆ^ *Y* is the projected number of admissions or deaths under the projected temperatures.

All analyses were conducted using the R software package (v3.0.0) (R Core Team 2013), and a two-sided *p*-value < 0.05 was considered statistically significant.

## Results

In 2010 the state of Rhode Island had a population of 1,052,567, with a median age of 39.4 years, about 48% male, and 81% white (see Supplemental Material, Table S2) (U.S. Census Bureau 2014). Between 2005 and 2012 there were approximately 1.6 million all-cause ED admissions. These patients had a mean age of 42.3 years, were predominantly female and white, and > 40% had public or no health insurance. Between 1999 and 2011 there were approximately 122,000 deaths. Those who died had a mean age of 74.9 years, were predominantly white, and were slightly more likely to be female than male. During April–October 1999–2012, the mean daily maximum temperature was 72.8°F and the mean dew point was 52.5°F (see Supplemental Material, Table S3).

**Figure 1:**
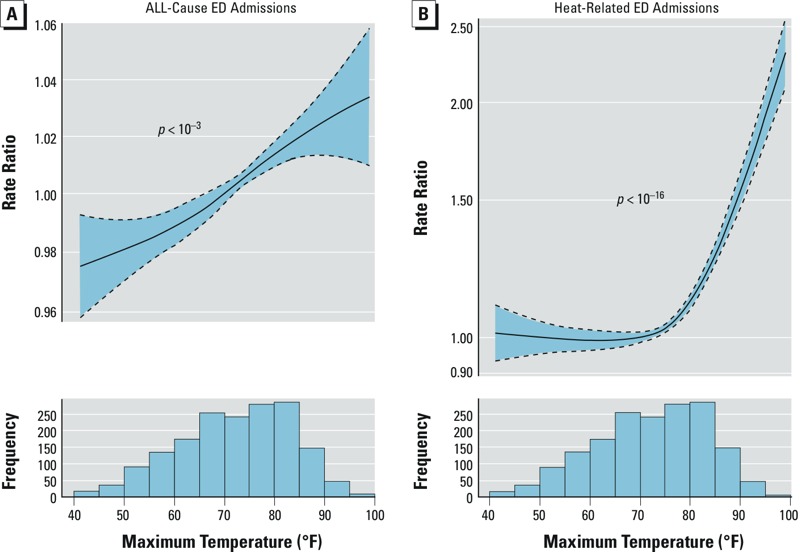
Natural spline fit showing the association between same-day maximum temperature and relative rate of all-cause (*A*) and heat-related (*B*) ED admissions in April–October 2005–2012 in Rhode Island. Maximum temperature was modeled using a natural cubic spline with 3 degrees of freedom and adjusted for day of week, federal holidays, O_3_, dew point (natural spline with 3 degrees of freedom), and temporal trends and seasonality (using a natural spline with 5 degrees of freedom per year). The dashed lines represent 95% CIs, and the *p*-value shown corresponds to the overall *p*-value comparing by ANOVA the full model to the same model without any terms for temperature.

*Morbidity.* In adjusted Poisson regression models, daily maximum temperature was statistically significantly associated with higher rates of both all-cause and heat-related ED admissions (Figure 1). The dose–response relationship for all-cause ED admissions was approximately linear (Figure 1A), with rates of all-cause ED admissions being about 1.2–1.4% higher per 10°F shift in maximum temperature (Table 1). In contrast, the association between daily maximum temperature and heat-related ED admissions shows a sharp increase in rate of admissions on days where maximum temperature exceeds 75°F (Figure 1B). For example, an increase in daily maximum temperature from 65 to 75°F was associated with a 3.3% (95% CI: 1.0%, 5.8%) higher rate of heat-related ED admissions, and an increase from 75 to 85°F was associated with a 23.9% (95% CI: 18.9%, 29.2%) higher rate of heat-related ED admissions. The association between maximum daily temperature and heat-related admissions was more pronounced (even at lower maximum daily temperatures) in a sensitivity analysis using an alternate, more specific definition of heat-related illness (see Supplemental Material, Table S4). Results were not statistically different in sensitivity analyses additionally adjusted for PM_2.5_ (ANOVA *p*-value = 0.74, data not shown).

**Table 1:** Estimated percent difference (95% CI) in rate of all-cause and heat-related ED admissions and all-cause deaths associated with specific increments in maximum daily temperature for April–October 2005–2012.

Temperature change (°F)	All-cause ED admissions (*n* = 1,626,105)	Heat-related ED admissions (*n* = 48,612)	All-cause deaths (*n* = 122,374)
60–70	1.2 (0.5, 1.8)*	0.6 (–2.4, 3.7)	1.7 (–0.9, 4.4)
65–75	1.4 (0.9, 1.9)*	3.3 (1.0, 5.8)*	2.4 (0.5, 4.3)*
70–80	1.4 (0.8, 2.0)*	10.8 (7.9, 13.8)*	3.1 (1.2, 5.2)*
75–85	1.3 (0.4, 2.2)*	23.9 (18.9, 29.2)*	4.0 (0.7, 7.3)*
80–90	1.2 (–0.3, 2.6)	38.5 (30.0, 48.0)*	4.7 (–0.8, 10.4)
Estimates are from overdispersed Poisson regression models adjusted for day of week, federal holidays, 8-hr maximum O_3_, dew point, and seasonal and long-term time trends. Heat-related ED admissions were defined as those with a primary or secondary discharge diagnosis of ICD-9: E900, 992, or 276.51.*p < 0.05.			

The association between maximum temperature and rates of all-cause ED admissions varied by age, with the strongest association observed for those ≥ 65 years of age (Table 2). The association with rates of heat-related ED admissions also varied by age, but with the strongest association observed among those 18–64 years of age. Point estimates for associations between daily maximum temperature and rate of all-cause ED admissions were consistently higher for males than females and for white versus non-white patients (see Supplemental Material, Table S5). However, daily maximum temperature > 70°F was significantly associated with higher rates of heat-related ED admissions in both whites and non-whites and in both sexes.

**Table 2:** Estimated percent difference (95% CI) in rate of all-cause and heat-related ED admissions associated with specific increments in maximum daily temperature for April–October 2005–2012, stratified by age group.

Temperature change (°F)	< 18 years	18–64 years	≥ 65 years
All-cause ED admissions	*n* = 240,836	*n* = 1,053,309	*n* = 331,960
60–70	1.7 (0.03, 3.4)*	0.3 (–0.5, 1.0)	3.6 (2.5, 4.8)*
65–75	2.3 (1.0, 3.6)*	0.3 (–0.3, 0.8)	4.3 (3.4, 5.2)*
70–80	2.5 (1.0, 4.0)*	0.3 (–0.4, 0.9)	4.4 (3.4, 5.4)*
75–85	2.2 (–0.2, 4.6)	0.4 (–0.7, 1.4)	3.8 (2.2, 5.5)*
80–90	1.8 (–2.1, 5.6)	0.5 (–1.2, 2.2)	3.1 (0.6, 5.7)*
Heat-related ED admissions	*n* = 4,716	*n* = 19,534	*n* = 24,362
60–70	–2.2 (–10.7, 7.0)	–1.3 (–5.8, 3.4)	2.9 (–1.3, 7.2)
65–75	–1.8 (–8.4, 5.3)	1.2 (–2.3, 4.9)	6.4 (3.1, 9.8)*
70–80	4.1 (–4.0, 12.9)	12.0 (7.5, 16.7)*	11.2 (7.2, 15.3)*
75–85	16.6 (2.6, 32.6)*	33.5 (25.4, 42.2)*	17.0 (10.5, 23.9)*
80–90	31.1 (6.8, 61.0)*	59.6 (44.7, 76.0)*	22.6 (11.8, 34.3)*
*p < 0.05.			

**Figure 2:**
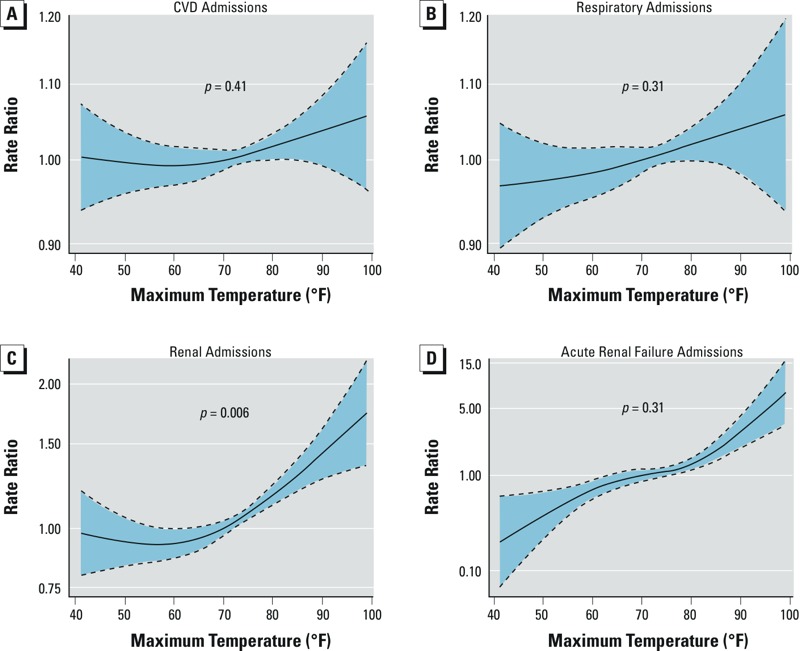
Natural cubic spline fit showing the association between same-day maximum temperature and relative rate of ED admissions for cardiovascular diseases (*A*), respiratory diseases (*B*), renal diseases (*C*), and acute renal failure (*D*) in Rhode Island, April–October 2005–2012. The modeling approach was analogous to that described in Figure 1. The dashed lines represent 95% CIs, and the *p*-value shown corresponds to the overall *p*-value comparing by ANOVA the full model to the same model without any terms for temperature.

Rates of ED admissions for respiratory disease, cardiovascular disease, acute renal failure, and asthma were not statistically significantly associated with daily maximum temperature overall (Figure 2; see Supplemental Material, Table S4 and Figure S1). However, higher maximum temperature was associated with higher rates of ED admissions for renal diseases overall and for acute renal failure in temperature increments of 75–85°F and 80–90°F. For example, an increase in mean daily temperature from 75 to 85°F was associated with a 19.5% (95% CI: 8.1%, 32.1%) higher rate of ED admissions for renal diseases and a 60.6% (95% CI: 18.0%, 118.5%) higher rate of ED visits specifically for acute renal failure. Additionally, an increase from 65 to 75°F was associated with a statistically significant negative association between asthma and maximum temperature.

**Figure 3:**
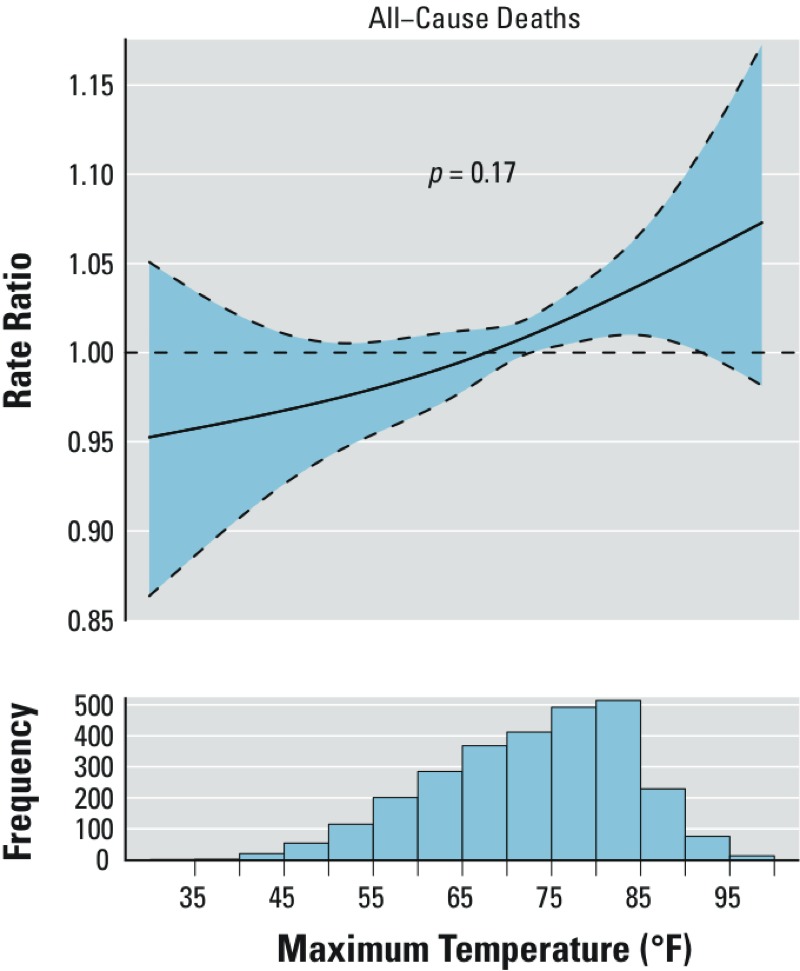
Natural cubic spline fit showing the association between same-day maximum temperature and relative rate of all-cause mortality in Rhode Island, April–October 1999–2011. The curved dashed lines represent 95% CIs, the horizontal dashed line indicates RR = 1.00, and the p-value shown corresponds to the overall p-value comparing by ANOVA the full model to the same model without any terms for temperature.

*Mortality.* The association between daily maximum temperature and rate of all-cause mortality was approximately linear (Figure 3). An increase in daily maximum temperature from 75 to 85°F was associated with a statistically significant 4.0% (95% CI: 0.7%, 7.3%) higher rate of all-cause deaths (Table 1). However, temperature overall was only a marginally statistically significant predictor of all-cause deaths after adjustment for other covariates (*p*-value = 0.17). Additionally adjusting for PM_2.5_ did not materially change the results (data not shown).

**Figure 4:**
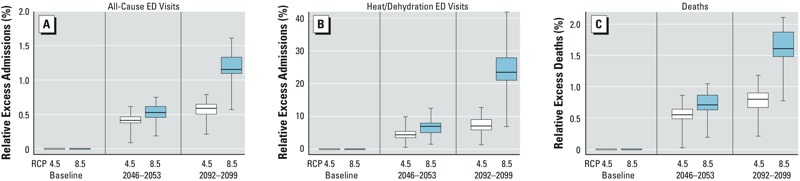
Box and whisker plot depicting the relative change in all-cause ED admissions (*A*), heat-related ED admissions (*B*), and deaths (*C*) projected to occur annually between April and October if the Rhode Island population of 2005–2012 were exposed to the maximum temperatures projected for 2046–2053 and 2092–2099 under two emissions scenarios. The heavy horizontal line in each box denotes the median, the limits of each box denote the 25th and 75th percentiles, and the whiskers denote the minimum and maximum of the estimates derived from the 42 models for RCP 4.5 and of the 41 models for RCP 8.5.

**Figure 5:**
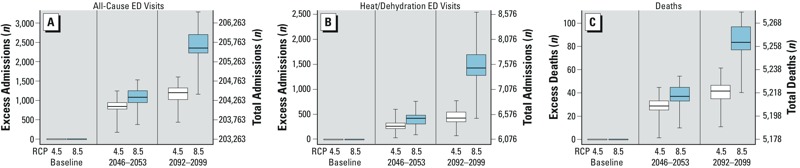
Box and whisker plots depicting the absolute change in all-cause ED admissions (*A*), heat-related ED admissions (*B*), and deaths (*C*) projected to occur annually between April and October if the Rhode Island population of 2005–2012 were exposed to the maximum temperatures projected for 2046–2053 and 2092–2099 under two emissions scenarios.

*Projected health effects of future temperatures.* Using the above associations between recent past daily maximum temperature and ED visits, we estimated the number of excess ED visits that would occur under the hypothetical scenario that the Rhode Island population of 2005–2012 were exposed to the higher maximum daily temperatures projected for 2046–2053 (centered at 2050), and 2092–2099 (centered at 2095). Under the RCP 8.5 emissions scenario and holding all other factors constant, we estimate that in this population there would be 1,084 (or 0.5%; range, 0.2%–0.8%) and 2,405 (or 1.2%; range, 0.6%–1.6%) additional all-cause ED admissions annually between April and October if faced with the higher temperatures projected for 2050 and 2095, respectively (Figures 4 and 5; see also Supplemental Material, Table S6). We likewise expect that there would be 411 (or 6.8%; range, 1.5%–12.5%) and 1,485 (or 24.4%; range, 6.9%–41.8%) more heat-related ED visits annually between April and October if this population were faced with the higher temperatures projected for 2050 and 2095, respectively. Fewer excess admissions would be expected under the lower-emissions RCP 4.5 scenario, as shown in Figures 4 and 5 (see also Supplemental Material, Table S6).

The relative increases in the number of deaths in this population each year between April and October are similar to what is projected for all-cause ED visits, reaching 1.6% (range, 0.8–2.1%) for the temperatures projected for the RCP 8.5 scenario for 2092–2099. However, because there are relatively few deaths per year in Rhode Island, the absolute numbers of excess deaths are smaller, with a mean of 84 (range, 40–109) excess deaths projected for the temperatures projected for the RCP 8.5 scenario for 2092–2099.

## Discussion

In Rhode Island we found that in April through October of 2005–2012, warmer temperatures were associated with higher rates of all-cause and heat-related ED admissions. The association between daily maximum temperature and rates of all-cause ED admissions was approximately linear, whereas the association with heat-related ED admissions increased sharply for maximum temperatures above ~ 75°F. When considering other specific causes of ED admissions, we found a pronounced association for admissions for renal diseases in general and acute renal failure in particular. Using data from 1999 to 2011 we also found that warmer temperatures tended to be associated with higher rates of all-cause mortality, that this association appeared to be approximately linear, and that it did not reach overall statistical significance likely because of the relatively small number of deaths observed in Rhode Island during the study period.

Our results regarding the association between heat and ED admissions are broadly consistent with those of prior studies, with modest associations typically observed for all-cause ED admissions and stronger associations observed for heat-related ED admissions (Basu et al. 2012; Lippmann et al. 2013; Schaffer et al. 2012; Williams et al. 2012a, 2012b). Our results are also consistent with a number of prior studies that have documented substantially higher rates of health care utilization for renal diseases in general, and acute renal failure in particular, associated with warm weather (Basu et al. 2012; Fletcher et al. 2012; Green et al. 2010; Gronlund et al. 2014; Hansen et al. 2008; Knowlton et al. 2009; Kovats et al. 2004; Li et al. 2011; Williams et al. 2012a).

There is less consistency across prior studies regarding the association between heat and rates of ED or hospital admissions for respiratory and cardiovascular diseases. For example, among U.S. Medicare beneficiaries, moderate heat has been associated with increased rates of hospital admissions for respiratory diseases, but not cardiovascular diseases (Anderson et al. 2013; Gronlund et al. 2014). Similar results have been documented in European studies (Kovats et al. 2004; Michelozzi et al. 2009; Wichmann et al. 2011). In contrast, other studies have found that heat is associated with rates of hospitalization for cardiovascular diseases (Knowlton et al. 2009; Lin et al. 2009; Ostro et al. 2010; Son et al. 2014), but not respiratory diseases (Basu et al. 2012; Knowlton et al. 2009; Son et al. 2014). Some of the heterogeneity in prior studies is likely attributable to considering broad categories of disease (e.g., the composite end point of cardiovascular diseases) as a single outcome, which may mask associations with more specific outcome definitions (Basu et al. 2012). In the present study, we estimated that between 2005 and 2012 each 10°F increase in maximum daily temperature was associated with approximately 2% higher rates of ED visits for both respiratory and cardiovascular diseases, but these associations did not reach statistical significance, suggesting that additional studies in larger data sets are needed.

Our finding that higher temperatures were associated with all-cause mortality is consistent with the large existing literature in this area. Specifically, we found that a 10°F increase in daily maximum temperature between 1999 and 2011 was associated with approximately a 2–4% higher rate of all-cause death, similar to our estimated association with all-cause ED admissions. The magnitude of this association is similar to those from some prior studies focusing on a range of temperatures rather than just extreme heat (Basu et al. 2008), but lower than that suggested by other studies (Curriero et al. 2002; Petkova et al. 2014). Indeed, the few prior studies that have considered both mortality and morbidity in the same population suggest that the association between heat and mortality would be expected to be stronger than the association between heat and morbidity (Kovats et al. 2004; Linares and Díaz 2008; Williams et al. 2012a, 2012b). The more modest association between heat and mortality observed in this study compared to other studies may be due to population adaptations or acclimatization in recent years (Davis et al. 2003; Petkova et al. 2014; Sheridan et al. 2008), or perhaps the typically moderate weather of Rhode Island.

Some prior studies suggest that increased rates of ED admissions due to warmer weather may be more pronounced among the elderly (Linares and Díaz 2008; Schaffer et al. 2012; Wichmann et al. 2011; Williams et al. 2012b), although other studies have failed to find important differences by age (Knowlton et al. 2009). Basu et al. (2012) noted that differences in associations according to age vary depending on the specific outcome being considered. We found that the association with maximum daily temperature and all-cause ED admissions was most pronounced among those ≥ 65 years of age, whereas the association with heat-related ED admissions was most pronounced among those 18–64 years of age. The suggestion that adults of working age might be most susceptible to heat-related ED visits is consistent with a study in North Carolina (Lippmann et al. 2013). Whether stronger associations in this age group reflect increased opportunities for exposure (e.g., through increased outdoor recreational or occupational activities), less careful attention to heat warnings, or are simply a function of the relatively lower baseline rate of ED admissions in this age group remains unclear.

We found that daily maximum temperature was more strongly associated with all-cause ED admission rates among whites and males (compared with non-whites and females, respectively), although when considering heat-related ED visits, results were more similar across race and sex. Prior studies have been mixed, with some finding differences by sex (Stafoggia et al. 2006) or race (Linares and Díaz 2008), and others not (Anderson et al. 2013; Basu et al. 2012). Differences in associations between heat and morbidity by sex and race might reflect differences in recreational or occupational activities, time spent outdoors, availability and use of air conditioning, socioeconomic differences, or differing health care utilization patterns, though noncausal explanations are also possible. Additional studies with detailed data on individual behaviors and characteristics would be needed to confirm or refute any of these hypotheses.

We estimated the number of all-cause ED admissions, heat-related ED admissions, and all-cause deaths if the Rhode Island population of 2005–2012 were exposed to the maximum daily temperatures projected for 2046–2053 and 2092–2099 under the RCP 8.5 and RCP 4.5 emissions scenarios. An equivalent interpretation of these estimates is the average number of ED admissions and deaths that would be seen each year between April and October of 2046–2053 and 2092–2099 under these two emissions scenarios if all other variables (e.g., population demographics, land use, air conditioning use, other adaptation strategies) were unchanged. Under both emissions scenarios, we estimate that there would be a greater number of all-cause and heat-related ED admissions annually between April and October if the current population of Rhode Island had been exposed to the higher maximum temperatures projected for the future. We estimate that the greatest relative increase would be for heat-related ED admissions, which would be up to 20% higher under temperatures projected by the RCP 8.5 emissions scenario for the end of the century. Projected increases in mortality are smaller, but with 25–90 annual excess deaths during the warm months of the year projected, these estimates are still of significant public health interest. Our estimates are broadly consistent with estimates previously published for morbidity (Lin et al. 2012; Ostro et al. 2012) and mortality (Knowlton et al. 2007; Peng et al. 2011; Wu et al. 2013), although direct comparison to those prior studies that have used different methods or older emissions scenarios and climate models is difficult.

As temperatures rise, populations will need to adapt to the heat. A previous study of 11 U.S. cities between 1973 and 1994 reported that, compared with southern cities, northern cities had a lower prevalence of air conditioning use and stronger associations between heat and adverse health outcomes (Curriero et al. 2002). Vulnerability to heat-related health effects varies by more than latitude. Previous studies have found that heat vulnerability also varies by socioeconomic status, age, social isolation, built environment, and air quality (Basu and Samet 2002; Curriero et al. 2002). Therefore future studies should include indices of heat vulnerability to better understand the subpopulations most at risk from heat-related morbidity and mortality.

This study has some important limitations. First, Rhode Island is a small state, and the available sample size may have limited our statistical power, especially for examining associations among subgroups where our estimates may be imprecise. Second, we used a kriging approach to estimate weather and air pollution levels at the geographic centroid of the state rather than at each individual’s place of residence. Although Rhode Island is a geographically small state, this may have resulted in some exposure misclassification. Third, our projections of the health impact of future climate change do not account for changes in population demographics, land use characteristics, other vulnerability factors, acclimatization, adaptation strategies or prevention efforts. Thus, these results can most easily be interpreted as what would be observed in Rhode Island had the population of today experienced the warmer temperatures projected for future years under two emissions scenarios. Fourth, in our analyses of the health impact of future temperatures we do not fully account for uncertainty in either our health effects estimates or uncertainty in climate models. For example, downscaled climate projections may contain materially important biases, especially near coastal areas and in the warm months of the year (Dixon et al. 2013). Fifth, we considered the health effects only of maximum daily temperature, although some studies suggest that mean or minimum daily temperature might also be important. However, prior studies suggest that results for maximum, minimum, and mean temperature are likely to be quite similar (Ebi et al. 2004; Gronlund et al. 2014; Williams et al. 2012b). Finally, we chose our analytic approach *a priori* based on our experience and common practice in prior studies, but did not explore in detail the sensitivity of our results to using alternative approaches with more or fewer degrees of freedom for smoothing functions of time, dew point, or temperature. We also did not explore other potential determinants of temporal variation in rates of ED admissions or death.

On the other hand, strengths of this study include the evaluation of the association between daily maximum temperature with both morbidity and mortality in a single population, consideration of a wide range of warm temperatures and among all ages, allowing for flexible temperature–response relationships, and projecting the number of additional ED admissions and deaths under two future emissions scenarios using > 40 climate models from the most recent CMIP5 climate change model projections.

In this study we found that moderate temperatures were associated with higher rates of ED admissions, heat-related ED admissions, and all-cause deaths. Currently, in Southern New England a heat advisory is issued when the heat index is forecast to reach 100–104°F for ≥ 2 hr. The results of the present study suggest that public health agencies may need to consider implementing educational campaigns and/or heat warning systems that reflect the potential health hazards of less extreme maximum daily temperatures. Indeed, public health interventions targeted at preventing heat-related illness at moderate temperatures may have a greater cumulative benefit than those warnings targeted only at extreme heat events, because the latter are by definition relatively rare.

## Conclusions

In summary, we found that maximum daily temperature was positively associated with the rate of all-cause and heat-related ED admissions between 2005 and 2012, and all-cause deaths between 1999 and 2011. In addition, our findings suggest that if all other characteristics are held constant, higher maximum daily temperatures projected for 2046–2053 and 2092–2099 will lead to higher morbidity and mortality in Rhode Island.

## Supplemental Material

(315 KB) PDF
