# Supplementary material for: Current and Projected Heat-Related Morbidity and Mortality in Rhode Island
Source: Environ Health Perspect. 2015 Aug 7;124(4):460–7. doi: 10.1289/ehp.1408826 (PMC4829994; doi:10.1289/ehp.1408826)
Supplement: (315 KB) PDF [file ehp.1408826.s001.acco.pdf]

Environ Health Perspect
DOI: 10.1289/ehp.1408826

Note to Readers: *EHP* strives to ensure that all journal content is accessible to all readers. However, some figures and Supplemental Material published in *EHP* articles may not conform to 508 standards due to the complexity of the information being presented. If you need assistance accessing journal content, please contact ehp508@niehs.nih.gov. Our staff will work with you to assess and meet your accessibility needs within 3 working days.

Supplemental Material

Current and Projected Heat-Related Morbidity and Mortality in Rhode Island

Samantha L. Kingsley, Melissa N. Eliot, Julia Gold, Robert R. Vanderslice, and Gregory A. Wellenius

Table of Contents

Table S1: CMIP5 Modeling centers (or groups) and model names.

Table S2: Characteristics of Rhode Island residents admitted to emergency departments (ED) in Rhode Island from 2005-2012, Rhode Island residents who died between 1999 and 2011, and the 2010 Rhode Island population.

Table S3: Descriptive statistics of the Rhode Island atmosphere April – October, 1999-2012.

Table S4: Estimated percent difference (95% confidence interval) in the rate of emergency department (ED) admissions for cardiovascular disease, respiratory diseases, asthma, renal diseases, acute renal failure, and heat in Rhode Island associated with specific increments in maximum daily temperature, from April-October, 2005-2012. Note that there are 1,626,105 total ED admissions during this period.

Table S5: Estimated percent difference (95% confidence interval) in the rate of all-cause and heat-related emergency department (ED) admissions associated with specific increments in maximum daily temperature for April – October of 2005-2012, stratified by sex and race.

Table S6: Observed and projected maximum temperatures and estimated numbers of all-cause emergency department (ED) admissions, heat-related ED admissions, and deaths projected to occur annually between April and October if the RI population of 2005-2012 were exposed to the maximum temperatures projected for 2046-2053 and 2092-2099 under two emissions scenarios, RCP 4.5 and RCP 8.5. Numbers in

parentheses denote the minimum and maximum estimates based on the multiple CMIP5 models applied for the two scenarios. N represents the number of each admission type in Rhode Island during the study period.

Figure S1: Natural cubic spline fit showing the association between same-day maximum temperature and relative rate of ED admissions for asthma in Rhode Island, April – October of 2005-2012. Modeling approach was analogous to that described in Figure 1. The dashed lines represent 95% and the p-value shown corresponds to the overall p-value comparing by ANOVA the full model to the same model without any terms for temperature.

Table S1: CMIP5 Modeling centers (or groups) and model names.

Modeling Center (or Group)	Institute ID	Model Name	RCP
Commonwealth Scientific and Industrial Research Organization (CSIRO) and Bureau of Meteorology (BOM), Australia	CSIRO-BOM	ACCESS1.0	4.5, 8.5
Beijing Climate Center, China Meteorological Administration	BCC	BCC-CSM1.1	4.5, 8.5
Canadian Centre for Climate Modelling and Analysis	CCCMA	CanESM2.1	4.5, 8.5
		CanESM2.2	4.5, 8.5
		CanESM2.3	4.5, 8.5
		CanESM2.4	4.5, 8.5
		CanESM2.5	4.5, 8.5
National Center for Atmospheric Research	NCAR	CCSM4.1	4.5, 8.5
		CCSM4.2	4.5, 8.5
Community Earth System Model Contributors	NSF-DOE-NCAR	CESM1(BGC)	4.5, 8.5
Centre National de Recherches Météorologiques / Centre Européen de Recherche et Formation Avancée en Calcul Scientifique	CNRM-CERFACS	CNRM-CM5	4.5, 8.5
Commonwealth Scientific and Industrial Research Organization in collaboration with Queensland Climate Change Centre of Excellence	CSIRO-QCCCE	CSIRO-Mk3.6.0.1	4.5, 8.5
		CSIRO-Mk3.6.0.2	4.5, 8.5
		CSIRO-Mk3.6.0.3	4.5, 8.5
		CSIRO-Mk3.6.0.4	4.5, 8.5
		CSIRO-Mk3.6.0.5	4.5, 8.5
		CSIRO-Mk3.6.0.6	4.5, 8.5
		CSIRO-Mk3.6.0.7	4.5, 8.5
		CSIRO-Mk3.6.0.8	4.5, 8.5
		CSIRO-Mk3.6.0.9	4.5, 8.5
		CSIRO-Mk3.6.0.10	4.5, 8.5
NOAA Geophysical Fluid Dynamics Laboratory	NOAA GFDL	GFDL-ESM2G	4.5, 8.5
		GFDL-ESM2M	4.5, 8.5
		GFDL-HIRAM-C360	8.5
Institute for Numerical Mathematics	INM	INM-CM4	4.5, 8.5
Institut Pierre-Simon Laplace	IPSL	IPSL-CM5A-LR1	4.5, 8.5
		IPSL-CM5A-MR1	4.5, 8.5
		IPSL-CM5A-LR2	4.5, 8.5
		IPSL-CM5A-LR3	4.5, 8.5
		IPSL-CM5A-LR4	4.5, 8.5
Japan Agency for Marine-Earth Science and Technology, Atmosphere and Ocean Research Institute (The University of Tokyo), and National Institute for Environmental Studies	MIROC	MIROC-ESM	4.5, 8.5
		MIROC-ESM-CHEM1	4.5, 8.5

Atmosphere and Ocean Research Institute (The University of Tokyo), National Institute for Environmental Studies, and Japan Agency for Marine-Earth Science and Technology	MIROC	MIROC5.1<br>MIROC5.2<br>MIROC5.3	4.5, 8.5<br>4.5, 8.5<br>4.5, 8.5
Max-Planck-Institut für Meteorologie (Max Planck Institute for Meteorology)	MPI-M	MPI-ESM-LR.1<br>MPI-ESM-LR.2<br>MPI-ESM-LR.3<br>MPI-ESM-MR-1<br>MPI-ESM-MR-2<br>MPI-ESM-MR-3	4.5, 8.5<br>4.5, 8.5<br>4.5, 8.5<br>4.5, 8.5<br>4.5<br>4.5
Meteorological Research Institute	MRI	MRI-CGCM3	4.5, 8.5
Norwegian Climate Centre	NCC	NorESM1-M	4.5, 8.5

Table S2: Characteristics of Rhode Island residents admitted to emergency departments (ED) in Rhode Island from 2005-2012, Rhode Island residents who died between 1999 and 2011, and the 2010 Rhode Island population.

**Characteristic**	**Total ED admissions, 2005-2012 (n = 1,626,105)**	**Total deaths, 1999-2011 (n = 122,374)**	**2010 Rhode Island Population[a] (n = 1,052,567)**
Age, mean ± SD	42.3 ± 24.4	74.9 ± 18.3	Median age = 39.4
Age group			
<18	14.8%	1.2%	20.4%
18-64	64.8%	18.9%	65.5%
65+	20.4%	79.9%	14.1%
Male, (%)	40.6%	46.3%	48.3%
White, (%)	72.3%	94.4%	81.4%
Health Insurance, (%)			
Private[b]	30.7%		
Medicare	26.0%		
Public, non-Medicare[c]	27.2%		
None	14.5%		
Other or unknown[d]	1.7%		

[a]From the 2010 US Census; [b]Includes Blue Cross, United, Harvard Pilgrim, Blue Chip, Tufts, HMO, "Commercial"; [c]Includes Medicaid and other government programs; [d]Includes worker's compensation

Table S3: Descriptive statistics of the Rhode Island atmosphere April – October, 1999-2012.

Atmosphere Characteristic	Mean ± SD	10th percentile	Median	90th percentile
Maximum temperature (°F)	72.8 ± 10.9	57.7	74.3	85.5
Average dew point (°F)	52.5 ± 12.4	34.1	54.5	67.2
Ozone (ppm)[a]	0.044 ± 0.015	0.027	0.043	0.063
$PM_{2.5}$ (μg/m^3)[b]	9.7 ± 6.5	3.81	7.74	18.31

[a]daily maximum 8 hour concentration; [b]daily mean concentration

Table S4: Estimated percent difference (95% confidence interval) in the rate of emergency department (ED) admissions for cardiovascular disease, respiratory diseases, asthma, renal diseases, acute renal failure, and heat in Rhode Island associated with specific increments in maximum daily temperature, from April-October, 2005-2012. Note that there are 1,626,105 total ED admissions during this period.

**ED Discharge Diagnosis**	**Temperature change (°F)**	**% change in rate (95% CI)**	**Overall p-value**
Cardiovascular Diseases (N = 64,580)	60-70	0.9 (-1.5, 3.4)	0.41
	65-75	1.6 (-0.3, 3.5)	
	70-80	2.1 (-0.1, 4.3)	
	75-85	2.3 (-1.2, 5.9)	
	80-90	2.3 (-3.4, 8.2)	
Respiratory Diseases (N = 53,044)	60-70	1.5 (-1.6, 4.7)	0.31
	65-75	1.8 (-0.6, 4.3)	
	70-80	2.0 (-0.8, 4.9)	
	75-85	2.0 (-2.5, 6.6)	
	80-90	1.8 (-5.4, 9.6)	
Asthma (N = 29,119)	60-70	-1.8 (-5.5, 2.1)	0.087
	65-75	-3.7 (-6.5, -0.7)*	
	70-80	-2.8 (-6.2, 0.6)	
	75-85	1.0 (-4.6, 7.0)	
	80-90	5.6 (-3.9, 16.1)	
Renal Diseases (N = 7,416)	60-70	7.1 (-0.4, 15.2)	0.006
	65-75	12.2 (6.2, 18.5)*	
	70-80	16.5 (9.3, 24.2)*	
	75-85	19.5 (8.1, 32.1)*	
	80-90	21.6 (3.6, 42.7)*	
Acute Renal Failure (N = 96)	60-70	15.5 (-10.6, 49.4)	0.31
	65-75	8.2 (-8.4, 27.9)	
	70-80	20.4 (-1.3, 47.0)	
	75-85	60.6 (18.0, 118.5)*	
	80-90	119.8 (34.6, 259.1)*	
Heat (N=1,161)	60-70	72.8 (31.1, 127.7)*	0.025
	65-75	152.7 (108.2, 206.7)*	
	70-80	247.0 (178.6, 332.0)*	
	75-85	331.7 (218.8, 484.6)*	
	80-90	401.3 (231.0, 659.2)*	

*p<0.05. Admissions are defined with ICD-9 codes as follows: Cardiovascular: 390-429 and 440-448; Respiratory: 480-487, 490-492, and 494-496; Asthma: 493; Renal: 580-589; Acute Renal Failure: 584; Heat: 992; E900.

Table S5: Estimated percent difference (95% confidence interval) in the rate of all-cause and heat-related emergency department (ED) admissions associated with specific increments in maximum daily temperature for April – October of 2005-2012, stratified by sex and race.

**Temperature change (°F)**	**Male (N = 660,509)**	**Female (N = 898,802)**	**White (N = 1,175,518)**	**Non-White (N = 450,413)**
All-Cause ED Admissions				
60-70	1.6 (0.7, 2.5)*	0.9 (0.0, 1.7)*	1.3 (0.6, 2.0)*	0.5 (-0.6, 1.7)
65-75	1.9 (1.3, 2.6)*	1.0 (0.4, 1.6)*	1.8 (1.3, 2.3)*	0.1 (-0.8, 0.9)
70-80	2.0 (1.3 2.8)*	0.9 (0.2, 1.7)*	1.9 (1.3, 2.5)*	0.0 (-1.0, 1.0)
75-85	1.9 (0.7, 3.1)*	0.7 (-0.5, 1.8)	1.7 (0.7, 2.7)*	0.4 (-1.2, 1.9)
80-90	1.8 (-0.2, 3.7)	0.4 (-1.5, 2.2)	1.3 (-0.3, 2.9)	0.8 (-1.7, 3.4)
Heat-Related ED Admissions				
60-70	-0.8 (-5.5, 4.0)	1.8 (-2.1, 5.8)	1.7 (-1.6, 5.0)	-4.7 (-10.3, 2.4)
65-75	0.9 (-2.7, 4.6)	5.0 (1.9, 8.2)*	4.1 (1.6, 6.7)*	-0.5 (-5.8, 5.0)
70-80	9.0 (4.5, 13.7)*	11.5 (7.7, 15.4)*	11.1 (7.9, 14.3)*	9.6 (3.0, 16.8)*
75-85	25.2 (17.3, 33.6)*	21.5 (15.1, 28.3)*	23.3 (17.9, 28.9)*	27.3 (15.5, 40.4)*
80-90	44.1 (30.2, 59.6)*	32.1 (21.2, 44.0)*	36.8 (27.5, 46.8)*	47.6 (26.6, 72.0)*

*p<0.05Note: There are a total of 1,626,105 admissions in RI during this time period. Some values for sex and race missing.

Table S6: Observed and projected maximum temperatures and estimated numbers of all-cause emergency department (ED) admissions, heat-related ED admissions, and deaths projected to occur annually between April and October if the RI population of 2005-2012 were exposed to the maximum temperatures projected for 2046-2053 and 2092-2099 under two emissions scenarios, RCP 4.5 and RCP 8.5. Numbers in parentheses denote the minimum and maximum estimates based on the multiple CMIP5 models applied for the two scenarios. N represents the number of each admission type in Rhode Island during the study period.

	**2005-2012**[a]	**2046-2053**		**2092-2099**	
		**RCP4.5**	**RCP8.5**	**RCP4.5**	**RCP8.5**
Mean Daily Maximum Temperature, °F	72.2	75.7 (72.9, 77.4)	76.7 (73.8, 78.7)	77.0 (74.1, 78.9)	82.4 (77.0, 86.3)
Difference of projected to reference period, °F		3.5 (0.8, 5.2)	4.6 (1.6, 6.5)	4.8 (1.9, 6.8)	10.2 (4.9, 14.1)
All-Cause ED Admissions, n	203,263	204,096 (203,442, 204,506)	204,347 (203,642, 204,793)	204,407 (203,698, 204,870)	205,668 (204,419, 206,542)
Difference of projected to reference period, n		833 (179, 1,243)	1,084 (379, 1,531)	1,144 (435, 1,606)	2,405 (1,156, 3,278)
Heat-Related ED Admissions, n	6,077	6,350 (6,110, 6,675)	6,488 (6,166, 6,837)	6,517 (6,149, 6,848)	7,562 (6,496, 8,618)
Difference of projected to reference period, n		273 (33, 598)	411 (90, 760)	440 (73, 771)	1,485 (419, 2,542)
All-Cause Deaths, n	5,178	5,206 (5,179, 5,222)	5,215 (5,188, 5,232)	5,218 (5,189, 5,239)	5,262 (5,218, 5,287)
Difference of projected to reference period, n		28 (1, 45)	38 (10, 54)	40 (11, 61)	84 (40, 109)

[a]1999-2011 was used as the reference period for analyses of all-cause deaths. Note: The CMIP5 models used are listed in Table S1.

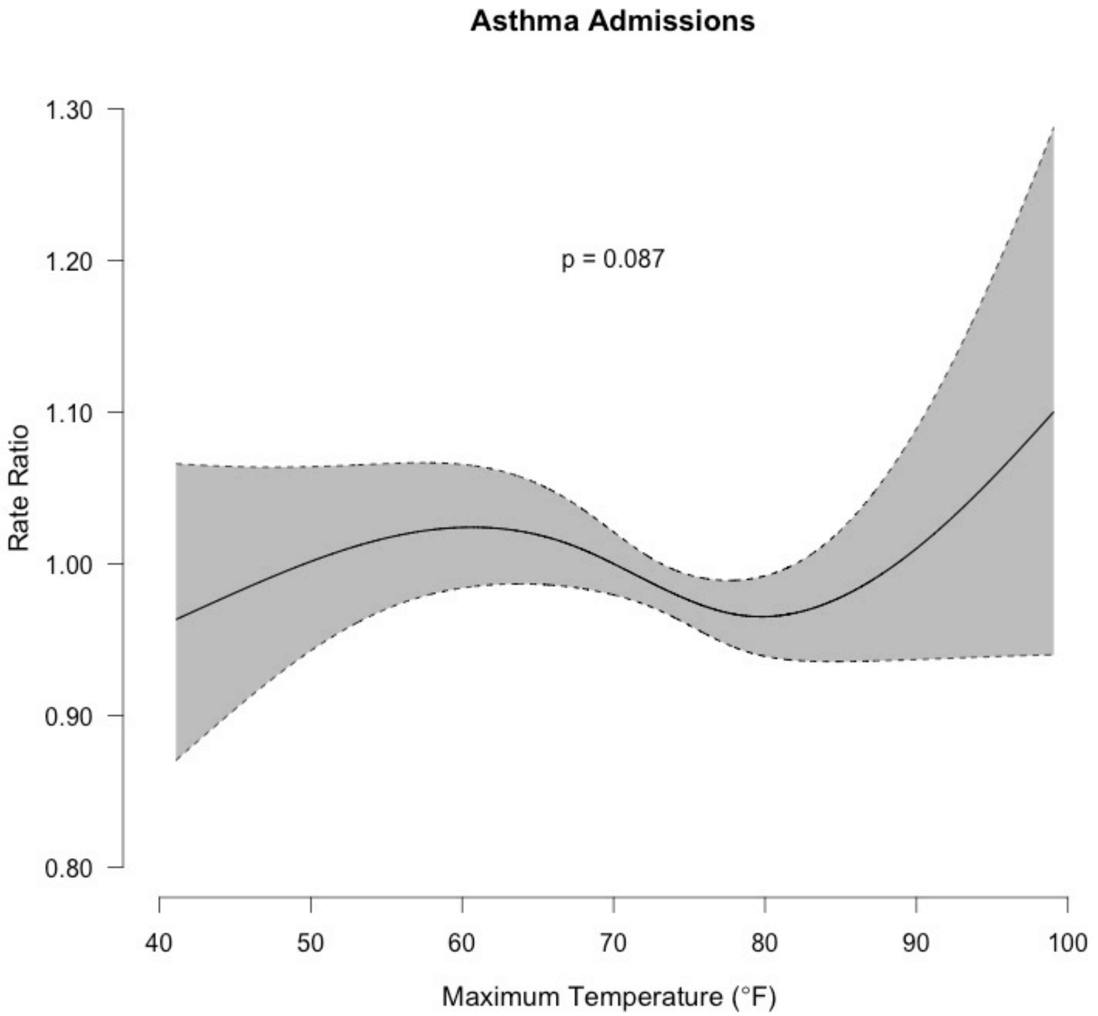

Figure S1: Natural cubic spline fit showing the association between same-day maximum temperature and relative rate of ED admissions for asthma in Rhode Island, April – October of 2005-2012. Modeling approach was analogous to that described in Figure 1. The dashed lines represent 95% and the p-value shown corresponds to the overall p-value comparing by ANOVA the full model to the same model without any terms for temperature.
